# Strategies for *in situ* laser heating in the diamond anvil cell at an X-ray diffraction beamline

**DOI:** 10.1107/S1600577513027434

**Published:** 2013-11-08

**Authors:** Sylvain Petitgirard, Ashkan Salamat, Pierre Beck, Gunnar Weck, Pierre Bouvier

**Affiliations:** a ID27, European Synchrotron Radiation Facility, 6 rue Jules Horowitz, BP 220, 38043 Grenoble Cedex 9, France; b Bayerisches GeoInstitut (BGI), University of Bayreuth, 95444 Bayreuth, Germany; c Lyman Laboratory of Physics, Harvard University, Cambridge, MA 02138, USA; d UJF-Grenoble 1/CNRS-INSU, Institut de Planetologie et d’Astrophysique de Grenoble (IPAG), 414 rue de la Piscine, 38000 Grenoble, France; e Commissariat à l’Energie Atomique (CEA), DPTA, 91680 Bruyères le Châtel, France; f Laboratoire des Materiaux et du Genie Physique, CNRS, Grenoble Institute of Technology, 3 parvis Louis Neel, F-38016 Grenoble, France

**Keywords:** laser heating, diamond anvil cell, CO_2_ laser, Nd:YAG pulsed laser, ID27 beamline

## Abstract

An overview of several innovations regarding *in situ* laser-heating techniques in the diamond anvil cell at the high-pressure beamline ID27 of the European Synchrotron Radiation Facility is presented. Pyrometry measurements have been adapted to allow simultaneous double-sided temperature measurements with the installation of two additional online laser systems: a CO_2_ and a pulsed Nd:YAG laser system. This reiteration of laser-heating advancements at ID27 is designed to pave the way for a new generation of state-of-the-art experiments that demand the need for synchrotron diffraction techniques. Experimental examples are provided for each major development. The capabilities of the double pyrometer have been tested with the Nd:YAG continuous-wave lasers but also in a time-resolved configuration using the nanosecond-pulsed Nd:YAG laser on a Fe sample up to 180 GPa and 2900 K. The combination of time-resolved X-ray diffraction with *in situ* CO_2_ laser heating is shown with the crystallization of a high-pressure phase of the naturally found pyrite mineral MnS_2_ (11 GPa, 1100–1650 K).

## Abstract 2

An overview of several innovations regarding *in situ* laser-heating techniques in the diamond anvil cell at the high-pressure beamline ID27 of the European Synchrotron Radiation Facility is presented.

## 1. Introduction   

Over the last decade the implementation of laser heating coupled with diamond anvil cell (DAC) experiments at large-scale facilities has lead to an increase in diversity in high-pressure research. The combined use of X-ray diffraction (XRD) with laser heating and high pressure provides an excellent probe for the study of phase transitions, melting, synthesis and structural analysis of systems over a very wide range of pressures and temperatures and has been used in various scientific domains such as chemistry (Young *et al.*, 2006 ▸), physics (Eremets *et al.*, 2004 ▸) and earth science (Murakami *et al.*, 2004 ▸).

In high-pressure research, two laser-heating techniques are commonly used and allow temperatures in excess of 4000 K to be generated for (i) metallic or conductive materials (Anzellini *et al.*, 2013 ▸) which require a wavelength in the micrometer range, or (ii) insulating materials (Zerr & Boehler, 1994 ▸) that need larger wavelengths of tens of micrometers to enable phonon propagation. On high-pressure beamlines, equipped with a laser-heating system, emission lines in the infrared (IR, λ = 1.064 µm) are usually generated using continuous-wave (CW) solid-state fibre lasers using either crystals of Nd:YAG (Nd^3+^-doped yttrium aluminium garnet) or Nd:YLF (Nd^3+^-doped yttrium lithium fluoride). More recently, time-resolved experiments have also been reported using a pulsed Nd:YAG laser at the Advanced Photon Source (APS, Argonne, USA) (Goncharov *et al.*, 2010 ▸) which can achieve even higher temperature. For insulating materials, an IR CO_2_ laser can be used, lasing at an order of magnitude higher at λ = 10.6 µm. A successful implementation is described at the high-pressure beamline of SPring-8 (Japan) (Murakami *et al.*, 2009 ▸), and there has been a high demand for similar laser facilities in recent years (Boulard *et al.*, 2011 ▸; Datchi *et al.*, 2012 ▸; Santoro *et al.*, 2012 ▸; Salamat *et al.*, 2013 ▸).

In the present article, improvements of temperature measurements and the installation of pulsed Nd:YAG and CO_2_ lasers at ID27 are described. These are aimed at increasing (i) the control of the temperature gradient within the sample, and (ii) the variety of samples that can be probed, making ID27 currently the only beamline where continuous pulsed Nd:YAG and CO_2_ laser heating can be carried out.

First we show how the existing optics set-up at ID27 has been modified to allow the measurement of temperature simultaneously from both sides of the sample on the same CCD camera. This allows the modulation of the power of the double laser-heating set-up to either provide homogeneous heating or to intentionally induce a thermal gradient within the sample. Both approaches are important for melting events or measuring thermal conductivity, respectively. We tested the latter by measuring the thermal gradient on a Fe foil through a pressure range of 63 to 180 GPa and temperature of 1500 to 2900 K with increasing the CW solid-state Nd:YAG laser power from each side of the sample alternately.

Second, we used the modified double spectro-radiometry set-up to install time-resolved experiments using a nanosecond-pulsed Nd:YAG laser on ID27. Preliminary results on heat propagation through the same Fe sample while decreasing the pressure from 180 to 100 GPa are presented.

The third implementation involves a CO_2_ laser-heating set-up to allow in particular the heating of minerals, oxides and organic materials. A final example is given with the *in situ* time-resolved transformation of the naturally found MnS_2_ mineral at a pressure of 11 GPa from 1150 to 1650 K using the newly installed CO_2_ laser.

## 2. Solid-state Nd:YAG laser heating and temperature measurements   

### 2.1. Beamline set-up and double-sided laser heating   

All the developments and X-ray measurements were carried out at the high-pressure beamline ID27 (ESRF, France). The source has a very high flux, owing to two in-vacuum undulators, over a large energy range, 20–70 keV, that can be easily tuned. A specific wavelength can be selected through a Si(111) channel-cut monochromator with an energy resolution of 10^−4^ (Δ*E*/*E*). For all the experiments presented in this article we used a monochromatic beam of *E* = 33 keV focused to a spot of size 3 µm × 2 µm using an achromatic Al_2_O_3_–Ir multi-layered Kirkpatrick-Baez (KB) double-mirror device (Mezouar *et al.*, 2005 ▸). The KB mirrors at ID27 also enable the laser spot to be scanned vertically and horizontally by the X-ray beam by tilting the corresponding bending mirrors. It is then possible to measure the thermal gradient with both X-ray diffraction and spectro-radiometry across the laser spot, or to make two-dimensional maps of the heated spot. This feature has been tested with an X-ray fluorescence set-up and described elsewhere (Petitgirard *et al.*, 2012 ▸). Diffraction patterns were collected with a 160 mm-diameter CCD detector from Marresearch, located at ∼200 mm from the sample. Debye rings were integrated with the *Fit2D* software (Hammersley *et al.*, 1996 ▸) and refined with *GSAS* (Toby, 2001 ▸).

**Figure 1:**
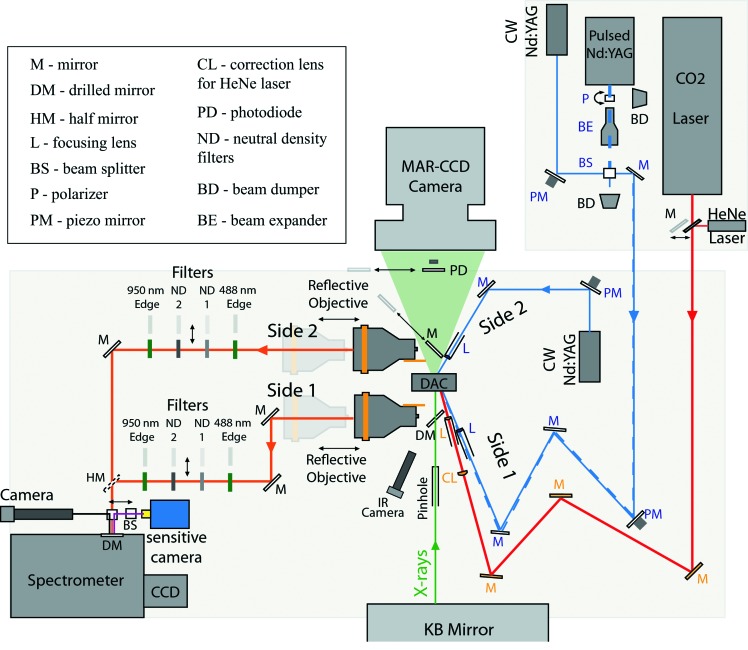
Schematic layout of the different laser-heating systems available at ID27. Red, blue and dashed blue lines indicate the incident CO_2_ and its HeNe alignment laser, Nd:YAG solid-state laser and pulsed Nd:YAG laser, respectively. In green are the incident X-ray beam and the X-ray cone of diffraction to the MAR CCD camera. In orange is the path for double-sided temperature measurement. Double-arrow signs refer to optics mounted on pneumatic stages.

For continuous double-sided laser heating, two CW Nd:YAG fiber lasers (IPG photonics) with λ = 1.064 µm and providing a maximum total power of 220 W are used to heat each side of the sample in the DAC (Fig. 1 ▸). The beam spot is in the fundamental transfer mode TEM_00_, or Gaussian profile; the beam has a very high quality with *M* ^2^ = 1.02 and the laser output power has a high stability of ±0.5%. UV-grade fused silica lenses with typical focusing distance of 70 mm are used to concentrate the laser power onto an area of typically 30 µm diameter. For each laser path, one of the mirrors is mounted on a piezo-actuator (Schultz *et al.*, 2005 ▸) (Fig. 1 ▸) allowing remote control of the laser spot while the X-rays are on.

### 2.2. Imaging of the sample and X-ray beam   

The light and image of the sample are collected using two Schwarzschild reflective objectives and transmitted to a 300 mm Acton spectrometer. The latter is equipped with a Pixis 100 CCD camera from Princeton Instruments (1340 × 100 pixels with a pixel size of 12.7 µm) with a quantum efficiency higher than 90% in the range 400–900 nm. The visualization of the sample is made using a mirror placed at the entrance of the spectrometer. The image is reflected back and deviated at 90° by a beam cube splitter, and then sent to a camera placed on a Navitar objective fixed perpendicular to the entrance of the spectrometer (Fig. 1 ▸). The mirror is laser drilled with two pinholes of diameter 50 µm separated by a distance of 1 mm, allowing two independent optical paths into the spectrometer (side 1 and side 2, see Fig. 1 ▸). To avoid any light contamination, a D-shape (or half) mirror has been installed; it is used as an edge to block the light from the other incoming path and physically separates the two paths (Fig. 1 ▸).

The two pinholes are also used as spatial references to align the X-ray beam at the same spatial position. For this procedure, the visualization of the sample is made with a fluorescence-sensitive coolSNAP-HQ^2^ camera, from Roper Instruments. This camera enables the visualization of the X-ray focal spot, using the X-ray fluorescence from the sample. Moving the translation stages below the two Schwarzschild objectives, the image is corrected in order to align the pinholes with respect to the image of the X-ray spot on both sides of the sample. Considering the magnification of the objectives (∼×18), the 50 µm pinholes correspond to a probed area of 7 µm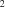 (*i.e.* 3 µm diameter) on the sample which is used to measure the temperature at the centre of the 30 µm hotspot created by the CW Nd:YAG lasers.

### 2.3. Double-sided temperature measurements   

**Figure 2:**
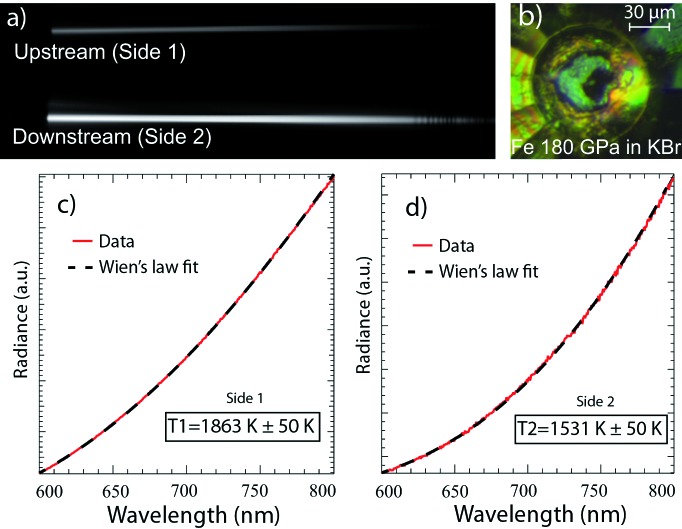
Typical temperature measurements on a Fe foil at 180 GPa. The Fe sample is laser heated from side 1 with a CW Nd:YAG laser, and the temperature is recorded on both sides simultaneously through the double pinhole entrance of the spectrometer. (*a*) Two-dimensional image of the CCD strip, and (*b*) picture of the Fe sample embedded in KBr at 180 GPa. (*c*), (*d*) Data integrated, calibrated and fitted with Wien’s law from 600 to 900 nm to retrieve the temperature from both sides (only the portion from 600 to 800 nm is shown here).

Temperatures are recorded simultaneously on both sides of the sample using the modifications describe above. The light entering the spectrometer through the two pinholes is diffracted by the gratings defining two regions of interest (ROIs) on the CCD strip (Fig. 2*a* ▸). The emission spectra are then integrated within the two ROIs giving the light intensity as a function of the wavelength. The spectrometer response is obtained with a calibrated tungsten lamp at 2500 K, a temperature close to all the results presented in this paper. The calibration procedure ensures that there is no light contamination from one side with respect to the other, and also that the measured temperature is the same on both sides for different power inputs from the tungsten filament. Once the emission spectra are corrected, temperatures can be calculated by fitting a portion of the curve, between 600 and 900 nm, using either a Planck or Wien function [Figs. 2(*c*) and 2(*d*) ▸]. The spectrometer response in such a case does not take into account the diamond as an optical component (Benedetti & Loubeyre, 2004 ▸) and special care should be taken for experiments aiming at reaching very high *T*. To overcome such problems, the spectrometer response can be made using a melting point of a well known metal, such as platinum, sealed in a DAC.

When carrying out laser heating, the Nd:YAG diffuse light enters both optical paths and can saturate the CCD chip. In order to attenuate the laser light prior to the entrance of the spectrometer, two optical blocking edge-short-pass filters at 950 nm from Semrock are installed. In addition, to avoid second-order UV reflection leading to a mis-diagnosis of the temperature determination at high temperatures (above 4500 K), we have placed two 488 nm RazorEdge long-pass filters in each optical path. In the present set-up, X-ray diffraction patterns can be recorded while the temperature is monitored from side 1 only. The mirror between the Schwarzschild and the DAC is drilled so the incoming X-ray beam can pass without modifying the light collection to the spectrometer. Recording X-ray diffraction patterns while measuring the temperature from both sides of the sample would be possible with the set-up by replacing the back mirror with a glassy carbon plate coated with a thin silver layer as found on other beamlines such as at APS (Argonne, USA) (Shen *et al.*, 2001 ▸) or at Petra III (Hamburg, Germany), and could be installed in the near future.

### 2.4. Evaluation of thermal gradients under pressure   

The separation of the incoming optical paths to the spectrometer enables the temperature to be measured on both sides of the sample simultaneously using a single CCD detector. It is then possible to evaluate the thermal gradients along the sample when carrying out laser heating. Such a feature becomes very important during melting experiments as the temperature on both sides of the sample should ideally be the same when the laser power is ramped up to achieve melting. The ability to monitor and control temperature on both sides of the DAC during heating can ensure homogeneous melting of the sample.

**Figure 3:**
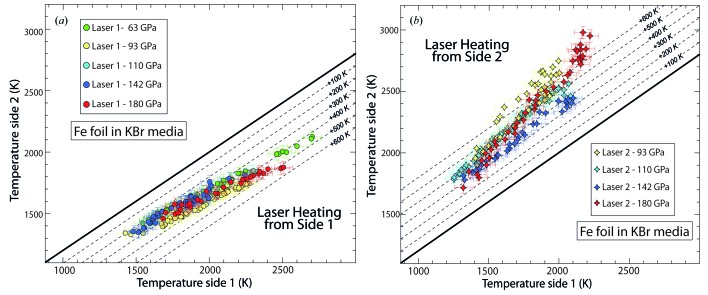
Simultaneous temperature measurements from both sides of the sample during laser heating on side 1 with laser 1 (*a*) and on side 2 with laser 2 (*b*) from 63 to 180 GPa. Temperatures measured from side 2 (*i.e.* upstream of the X-rays, see Fig. 1 ▸) are reported on the vertical axis, and temperatures measured from side 1 are reported on the horizontal axis. Circles indicate temperature measurements obtained during laser heating from side 1 only (*a*) and diamonds represent the temperature measured on both sides of the samples while laser heating from side 2 only (*b*). The dark thick line is representative of an absence of gradient in the sample, *i.e.* equal temperature from both sides of the sample. The dashed line refers to isotherms, shifted from the thick line.

However, for some experiments, inducing a thermal gradient and measuring its evolution with increasing *T* or *P* can be of prime importance. For example, the thermal conductivity of iron has been calculated at high pressure based on the measurement of the thermal gradient in the Fe sample in the DAC (Konopkova *et al.*, 2011 ▸). By using the temperature difference across a Fe foil up to 70 GPa in argon, it was possible to measure the thermal conductivity using further two-dimensional modelling. Such a calculation requires the evaluation of the thickness of the sample and the amount of argon on both sides of the sample. It also requires the estimation of the thermal conductivity of the Ar medium, to model and retrieve the thermal conductivity of the metal foil under pressure. We carried out a similar approach to test the newly installed spectro-radiometry set-up (§2.2–§2.3). A Fe foil of about 1 µm was sandwiched between two KBr pellets in a 35 µm-diameter sample chamber drilled in a 15 µm-thick rhenium gasket and pressurized from 63 to 180 GPa [Figs. 2(*b*) ▸ and 3 ▸]. At each pressure step the sample was heated on side 1 first (Fig. 1 ▸) and the thermal gradient was calculated using the double-sided temperature measurement. During this procedure the laser power was ramped up from a temperature of 1500 K to 2600 K and back as shown in Fig. 3(*a*) ▸. The same procedure was then repeated on side 2 with laser 2 (Fig. 1 ▸) with temperature ranging from 1600 K to 2900 K (Fig. 3*b* ▸). Fig. 3 ▸ illustrates the high precision obtained for temperature measurements when ramping up and down the laser, but also when switching from laser 1 to laser 2.

When heating on side 1 with a low laser power up to 1700 K, the measured temperature on side 2 is 300 K less at 1400 K (Fig. 3*a* ▸). Switching laser to side 2, we observed a similar effect with a slightly higher temperature difference of 350 K. In both cases this difference increases as the laser power was ramped up following a similar linear trend up to 2500 K with values of 450 K and 500 K for the thermal gradient on side 1 and on side 2, respectively.

This emphasizes that the thermal conductivity *k* of solid iron may have a linear dependence with increasing temperature in the *P*–*T* domain investigated (60–180 GPa and 1500–2500 K). The pressure dependence, or change in density from 60 to 180 GPa, seems to be rather small and counterbalanced by the change of *k* in solid iron as the trend stays similar at each pressure points.

When laser heating from side 1, the measured temperatures have a fairly low scattering along this linear trend from pressures of 63 (only investigated from side 1) to 180 GPa. Measurements from side 2 are more scattered. When lowering the laser power, the measured temperatures do not follow the same path observed when ramping up and describe a loop. This could be attributed to a less efficient insulation from this side of the sample. Nevertheless, the general trend is similar to the one observed from side 1 following a line from 93 to 180 GPa. Above 2500–2600 K at the highest pressure the measured temperatures seem to deviate from the linear trend in both cases [Figs. 3(*a*) and 3(*b*) ▸] and the calculated differences increase faster above 2600 K. Further investigation and similar calculation, as described by Konopkova *et al.* (2011 ▸), should be carried out to obtain values of *k* at higher pressures for a better understanding of the processes occurring at higher temperatures.

## 3. Pulsed YAG laser set-up   

The determination of thermal conductivity *k* of materials under extreme conditions is relevant in many fields of research such as material science and planetary science. For instance, values for the heat flow at the core–mantle boundary have doubled or even tripled in the recent years. Very recently, *ab initio* calculations on the thermal conductivity of iron and iron alloys at core conditions have been successfully carried out (Pozzo *et al.*, 2012 ▸), but so far no experimental data are available. Such data may also help to model the intensity and evolution of the geomagnetic field (Olson *et al.*, 2013 ▸) and the crystallization age of the inner core (Deguen & Cardin, 2011 ▸).

The thermal diffusivity *D* related to *k* through the following expression, *D* = *k*/(ρ*C*_P_) (with *D* the thermal diffusivity, *k* the themal conductivity, ρ the density and *C*_P_ the heat capacity), can be calculated using the double spectro-radiometry set-up described previously (§2.2–§2.3) in a fast-pulsed mode. The method, comparable with the Angstrom method established recently in multi-anvils (Manthilake *et al.*, 2011 ▸), requires a periodic temperature signal (or pulse) to be induced on one side of a sample and collecting the delay of propagation of the heat pulse from both sides of the sample. In the DAC a similar technique has been used in the reflection mode, called the transient heating technique (THT) (Beck *et al.*, 2007 ▸). In the THT, a nanosecond-pulsed Nd:YAG laser is used to heat a very thin Ir foil (100 nm), assuming that the foil returns to room temperature immediately after the pulse. The collected emission light, from the same side as the laser, is then related only to the different insulating material, MgO, KBr or KCl, and used to calculate their thermal diffusivity up to 25 GPa. A recent study has reported the thermal conductivity of argon up to 50 GPa (Goncharov *et al.*, 2012 ▸) by combining a microsecond laser with an intensified fast-gated camera. This study makes use of a modified THT technique with laser heating and collection of the emitted light from the same side of the sample and two-dimensional calculation to retrieve the thermal conductivity. In these two studies the metal foil is used only as a heater and the material of interest is the pressure-transmitting medium as well as insulator material.

Our developments on the set-up at ID27 were used for time-resolved temperature measurements. We followed the propagation of heat pulses through the Fe sample (§2.4) from 180 to 100 GPa, while the thickness of the sample remains quasi-identical through decompression. Thermal conductivity of a given material can be then addressed using this method as all the parameters are constrained, *i.e.* thickness of the sample, delay and temperature between heat maxima on each side of the sample.

### 3.1. Pulse detection and temperature measurements   

In this set-up, the laser is an IR diode-pumped Q-switched laser from Spectra-Physics (1064 nm, YAG) with a high repetition rate from 1 to 150 KHz. The beam has a diameter of 300 µm with a low divergence of 4.5 mrad and requires the use of a beam-expander for tighter focusing on the sample using a 70 mm focal dielectric lens (Fig. 1 ▸). The main characteristics of the pulse are a high energy per pulse >140 µJ, a pulse width of 8 ns at 20 KHz and 19 mA and also an excellent pulse-to-pulse stability. The pulse energy was finely tuned by a motorized polarizer in order to keep the other pulse parameters constant (pulse repetition rate, pulse width and jitter). The thermal emissions on both sides of the sample are transmitted to the Acton spectrometer, and for time-resolved experiments the Pixis 100 CCD camera is replaced by a fast-gated intensified CCD camera iStar from Andor Technology with a minimum gating of 2 ns. The intensifier is a GEN 3 version with a quantum efficiency higher than 20% in the range between 350 nm and 750 nm, and drops above 750 nm. The peaking time between each collection can be as low as 5 ps, creating an overlapping of the measurements. In our case we chose a minimum collecting time of 2 ns. The measurements correspond to an averaging over approximately 50000 cycles equivalent to 100 µs collection time. The time between each measure was set to 500 ps. In the set-up the iStar camera triggers the laser emission using the TTL signal from the gating of the CCD.

### 3.2. Results   

**Figure 4:**
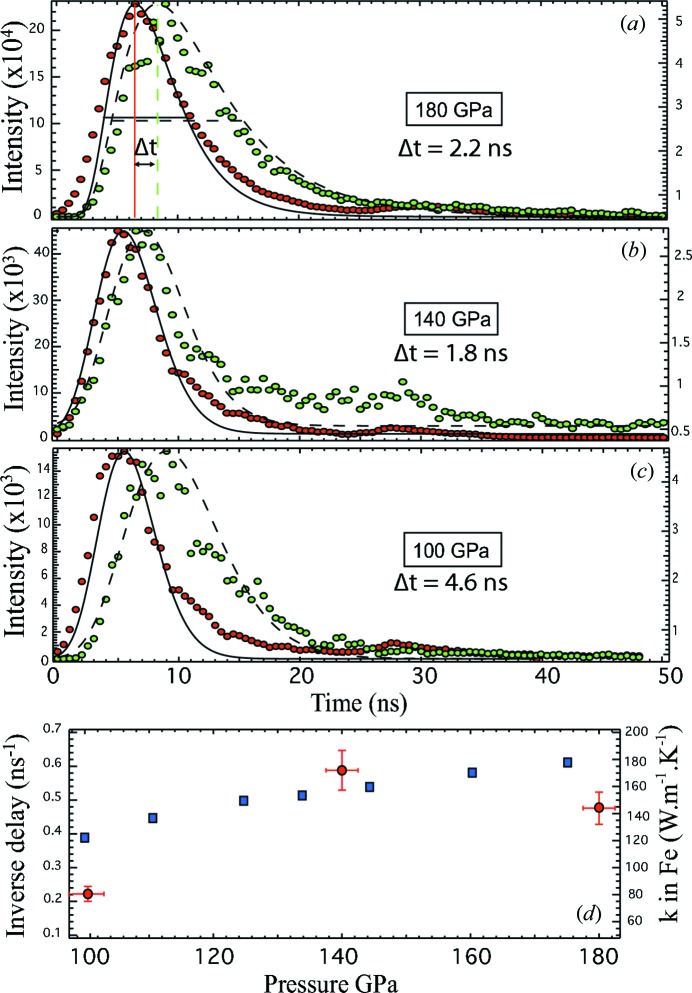
Pulse laser-heating results showing the time delay of the collected light from 180 to 100 GPa. The pulse laser is focused on the sample from side 1 (see Fig. 1 ▸). (*a*), (*b*), (*c*) Plots of near-IR intensity collected from the sample *versus* time (in ns) at 180 GPa, 140 GPa and 100 GPa, respectively. Red points are collected from side 1 (laser-heating side) and green points from side 2. The black curves are the pseudo-Voigt fits for the curve obtain from side 1, with black dashed curves for side 2. The time delay is calculated using the position of the two FWHMs for each side as illustrated in (*a*). (*d*) Plots of the inverse delay (in ns^−1^) (left scale) at the three investigated pressures (red circles); also reported are the results for thermal conductivity of molten iron (right-hand scale) as a function of pressure (blue squares) (Pozzo *et al.*, 2012 ▸).

As a preliminary test, the sample described in §2.4 was used with the pulsed laser during decompression from 180 to 100 GPa. Fig. 4 ▸ shows the delay of the light in the near-IR (or heat pulse) between the two sides of the sample at the different pressures. The measured temperature was around 3000 K, with, however, incertitudes of ±500 K; this should be solved using an intensifier with a sensitivity ranging from 400 to 900 nm. The light collected by the iStar camera corresponds to the heat propagation induced by the laser pulse. It reached the CCD from the laser-heating side first and a few nano­seconds later the other maxima reached the CCD. The heat pulse propagation can be fitted using a pseudo-Voigt function for each side [Figs. 4(*a*)–4(*c*) ▸]. The time delay between the two heat maxima was calculated using the difference between the positions of the full width at half-maximum (FWHM) of the heat curves. When decreasing pressure, the delay observed between the two FWHM of the heat pulse stays very similar at 140 GPa, and increases at 100 GPa as illustrated in Fig. 4 ▸. Such an effect could be attributed to (i) the increase of thickness of the sample while pressure decreases and (ii) the decrease of the thermal conductivity *k* with the decrease of the pressure (Pozzo *et al.*, 2012 ▸).

The recovered sample was measured with a scanning electron microscope and its thickness was estimated to be around 0.4 µm ± 0.1 µm. Using the following approximation: *L* ^2^ ≃ *Dt* (with *L* the thickness of the sample, *D* the thermal diffusivity and *t* the time delay in ns), we can roughly calculate the time delay in such a thin sample and we obtained values between 1 and 10 ns. We considered values for *D* between 2.5 × 10^−5^ m^2^ s^−1^ (room conditions) and 10^−4^ m^2^ s^−1^. The first value for *D* can be found in tables for molten iron and the second value is four times higher than at room conditions. The latter is of the same order of changes as *k* between the room *P* value and values from calculation in molten iron (Pozzo *et al.*, 2012 ▸) at core conditions. Also noticeable is the change in the inverse time delay decreasing by about 50% with an inverse delay of 0.45 ns at 180 GPa and 0.22 ns at 100 GPa (Fig. 4*d* ▸). A similar decrease of 50% is also found for *k* in molten iron with a decrease from 180 to 120 W m^−1^ K^−1^ between 180 and 100 GPa, respectively (Fig. 4*d* ▸). Values from calculations in molten iron and for the inverse time delay in solid iron are co-plotted in Fig. 4(*d*) ▸, illustrating the similarities in both trends. The point at 140 GPa is higher but laser heating was not as stable as for the two other points. Better calculations should be achieved by measuring the sample dimensions precisely before and after the experiment. For instance, it would be much clearer with a thicker sample of 1–1.5 µm at high pressure, which in terms of time delay would give values higher than 10 ns.

During these experiments we used the X-rays to measure the pressure in the sample chamber, but more importantly as a spatial reference to keep the pinholes on both sides of the sample at the exact same position. Also, using the absorption of the X-rays through the sample chamber, the thickness of the foil can be roughly estimated.

## 4. *In situ* CO_2_ laser-heating set-up   

Laser heating of insulating materials is scarce due to the many challenges related to the installation of a CO_2_ laser. To circumvent these problems, it was previously common to mix the sample with a metal-dopant that acts as a heater when coupling with the CW Nd:YAG laser source (Salamat *et al.*, 2009 ▸). However, this approach can result in a number of complications when attempting to understand the thermodynamics of the reaction along with the possibility of making various undesired analogous metallic compounds. This makes CO_2_ laser heating indispensable for carrying out dopant-free experiments involving organic and inorganic insulating systems.

### 4.1. Optical layout and correction lens   

The newly installed CO_2_ laser (Firestar f200 from Synrad) at ID27 is a Z RF resonator type, with a beam waist positioned at the output coupler with a diameter of 4 mm (1/*e* ^2^) that can deliver a maximum output power of 200 W. The absorption by SiO_2_ at this wavelength of light makes glass objectives unusable and the laser is focused with a plano convex ZnSe focusing lens of *f* = 75 mm located at 185 cm from the waist. The beam diameter at the lens is 8.6 mm and is focused to a calculated 60 µm spot size. We assume TEM_00_ mode for the beam profile of the laser.

In contrast to the use of solid-state lasers, the CO_2_ laser-heating technique provides a greater penetration depth, often equivalent to the sample thickness (tens of micrometers) and thus removing the need for double-sided heating. To minimize the procedure time for alignment as well as having a method for permanently identifying the path of the IR laser line we have installed a HeNe (λ = 633 nm) laser sitting coaxially in the path of the IR (λ = 10.6 µm) source. This allows for a visible reference line to be detected and adjusted during the alignment procedure. The HeNe laser line is introduced into the IR optical path using a motorized flipping mirror and the two laser lines are aligned over a distance of 3 m to ensure a greater precision of alignment. To correct for the ZnSe chromatism and waist discrepancies between the CO_2_ and HeNe lines a correction lens is installed, positioned at 180 mm before the ZnSe lens. It focuses the visible HeNe beam on the same plane as the IR laser. The correction lens is mounted on a flipping mirror and is controlled by the same control unit as the flipping mirror that introduces the HeNe line into the optical path. Both flipping mirrors are synchronized and thus prevent the danger of coupling the SiO_2_ optics with the incoming IR radiation.

The next challenge for the optical set-up was the reproducibility for alignment of the IR laser. When using the Nd:YAG lasers (λ = 1.064 µm), a standard CCD camera has a wide enough spectral range to pick up the signal and the 1 µm light can be visualized. This is clearly not the case when using the CO_2_ (λ = 10.6 µm) laser making the IR light invisible to the standard CCD. An extra hurdle was that the visualization set-up for Nd:YAG relies on back illumination and provides a field of view (FOV) of up to 500 µm in the DAC. Therefore, to make the alignment procedure easier we installed a CCD camera with a FOV of 3.2 mm and a working distance of 100 mm (Fig. 1 ▸). Although also limited in only viewing up to the near-IR range of the full spectrum of light, the wider field of view enables visualization of the diffusion of the sample itself or more conveniently diffusion of the metal gasket inner rough surface around the sample which then generates a signal that can be monitored. Once the location of the laser spot is roughly known, then a horizontal–vertical raster scan of the CO_2_ laser, with a step size smaller than the sample (typically 20 µm steps), is conducted and the absorption of the radiation by the sample is then observed by the coolSNAP-HQ^2^ camera downstream at very low power.

### 4.2. Time-resolved experiment with CO_2_ laser   

We carried out CO_2_ laser heating of the naturally found mineral MnS_2_ *in situ* with synchrotron diffraction techniques. The sample, a natural single crystal, was chipped and ground into a fine powder before being pressed into a pellet of dimensions 30 µm × 30 µm × 15 µm. The sample was elevated on a tripod of ruby spheres measuring 5–6 µm in diameter and neon was used as the thermal insulator and pressure-transmitting medium. The pressure was calibrated using the re-calibrated ruby fluorescence scale (Dewaele *et al.*, 2008 ▸). Laser heating was carried out *in situ* at ID27 and the annealing process monitored with time-resolved X-ray diffraction. Temperature was calculated from the thermal emission measurements collected from the hotspot of the CO_2_ laser irradiation and fitted with a Wien function (see §2.2).

**Figure 5:**
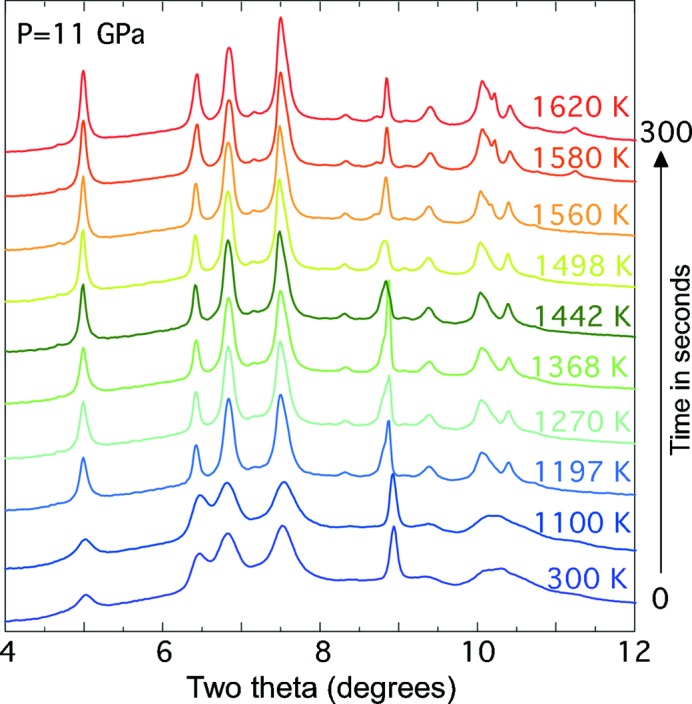
An *in situ* X-ray diffraction stack plot of the mineral MnS_2_ during CO_2_ laser heating. Temperature measurements were taken every 30 s and the full time lapse of this plot is 5 min.

The ambient phase of MnS_2_ is 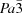 and by 11 GPa we observed that the system undergoes pressure-induced dis­ordering with broad featureless peaks (Fig. 5 ▸). Laser heating was carried out whilst using the synchrotron X-rays as a probe for determining the onset of a phase transition and full completion to a high-density phase. Fig. 5 ▸ shows the full transformation of the MnS_2_ 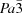 to a new phase following laser heating over a total period of 5 min with the laser power slowly increased from 35 to 60 W. This equates to a calculated temperature range of 1220–1600 K. Full details of this experiment and structural analysis are given elsewhere (Kimber *et al.*, 2013 ▸).

## 5. Conclusion   

We have presented several innovations for *in situ* laser heating in diamond anvil cells coupled with X-rays analysis. The improvements in the pyrometry measurement are vital for a wide range of experiments, in particular in estimating the thermal gradient by measuring simultaneously the temperature from both sides of the sample. The pulsed heating experiment demonstrates the feasibility of fast kinetic analysis at ID27. Some effort to synchronize X-ray pulses in the kHz regime and isolate a single bunch of a few hundred picoseconds in order to probe the sample using nanosecond-pulsed lasers are under progress at large-scale facilities. For example, a chopper has recently been designed to isolate a single bunch of synchrotron radiation of duration 150 ps (Cammarata *et al.*, 2009 ▸), and has been used for pump–probe experiments (Bourgeois *et al.*, 2003 ▸). In a cumulative mode and using the X-ray pulse as the probe and the laser as the pump it should be possible to synchronize XRD with the pulsed laser data in such a fast regime. Such experiments will surely make the link with future shock experiments that will take place at synchrotron sources and at even higher speeds at facilities such as free-electron lasers. The developments presented in this paper for double-sided pyrometry together with the successful installation of a CO_2_ laser offer capabilities to probe samples with wide compositional diversities used by the different communities involved in high-pressure science. 

## References

[bb1] Anzellini, S., Dewaele, A., Mezouar, M., Loubeyre, P. & Morard, G. (2013). *Science*, **340**, 464–466. 10.1126/science.1233514 23620049

[bb2] Beck, P., Goncharov, A. F., Struzhkin, V. V., Militzer, B., Mao, H. K. & Hemley, R. J. (2007). *Appl. Phys. Lett.* **91**, 181914.

[bb3] Benedetti, L. R. & Loubeyre, P. (2004). *High Press. Res.* **24**, 423–445.

[bb4] Boulard, E., Gloter, A., Corgne, A., Antonangeli, D., Auzende, A. L., Perrillat, J. P., Guyot, F. & Fiquet, G. (2011). *Proc. Nac. Acad. Sci. USA*, **108**, 5184–5187. 10.1073/pnas.1016934108 PMC3069163 21402927

[bb5] Bourgeois, D., Vallone, B., Schotte, F., Arcovito, A., Miele, A. E., Sciara, G., Wulff, M., Anfinrud, P. & Brunori, M. (2003). *Proc. Natl Acad. Sci. USA*, **100**, 8704–8709. 10.1073/pnas.1430900100 PMC166376 12847289

[bb6] Cammarata, M., Eybert, L., Ewald, F., Reichenbach, W., Wulff, M., Anfinrud, P., Schotte, F., Plech, A., Kong, Q. Y., Lorenc, M., Lindenau, B., Rabiger, J. & Polachowski, S. (2009). *Rev. Sci. Instrum.* **80**, 015101. 10.1063/1.3036983 19191457

[bb7] Datchi, F., Mallick, B., Salamat, A. & Ninet, S. (2012). *Phys. Rev. Lett.* **108**, 125701. 10.1103/PhysRevLett.108.125701 22540597

[bb8] Deguen, R. & Cardin, P. (2011). *Geophys. J. Int.* **187**, 1101–1118.

[bb9] Dewaele, A., Torrent, M., Loubeyre, P. & Mezouar, M. (2008). *Phys. Rev. B*, **78**, 104102.

[bb10] Eremets, M. I., Gavriliuk, A. G., Trojan, I. A., Dzivenko, D. A. & Boehler, R. (2004). *Nat. Mater.* **3**, 558–563. 10.1038/nmat1146 15235595

[bb11] Goncharov, A. F., Prakapenka, V. B., Struzhkin, V. V., Kantor, I., Rivers, M. L. & Dalton, D. A. (2010). *Rev. Sci. Instrum.* **81**, 113902. 10.1063/1.3499358 21133481

[bb12] Goncharov, A. F., Wong, M., Dalton, D. A., Ojwang, J. G. O., Struzhkin, V. V., Konopkova, Z. & Lazor, P. (2012). *J. Appl. Phys.* **111**, 112609.

[bb13] Hammersley, A. P., Svensson, S. O., Hanfland, M., Fitch, A. N. & Hausermann, D. (1996). *High Press. Res.* **14**, 235–248.

[bb14] Kimber, S., Salamat, A., Evans, S., Jeschke, H., Muthukumar, K., Tomic, M., Salvat-Pujol, F., Valenti, R., Kaisheva, M. V., Zizak, I. & Chatterji, T. (2013). arXiv:1305.7119 [cond-Mater.str-el]. 10.1073/pnas.1318543111 PMC3986163 24706831

[bb15] Konopkova, Z., Lazor, P., Goncharov, A. F. & Struzhkin, V. V. (2011). *High Press. Res.* **31**, 228–236.

[bb16] Manthilake, G. M., de Koker, N., Frost, D. J. & McCammon, C. A. (2011). *Proc. Natl Acad. Sci.*, **108**, 17901–17904. 10.1073/pnas.1110594108 PMC3207700 22021444

[bb17] Mezouar, M., Crichton, W. A., Bauchau, S., Thurel, F., Witsch, H., Torrecillas, F., Blattmann, G., Marion, P., Dabin, Y., Chavanne, J., Hignette, O., Morawe, C. & Borel, C. (2005). *J. Synchrotron Rad.* **12**, 659–664. 10.1107/S0909049505023216 16120991

[bb18] Murakami, M., Asahara, Y., Ohishi, Y., Hirao, N. & Hirose, K. (2009). *Phys. Earth Planet. Inter.* **174**, 282–291.

[bb19] Murakami, M., Hirose, K., Kawamura, K., Sata, N. & Ohishi, Y. (2004). *Science*, **304**, 855–858. 10.1126/science.1095932 15073323

[bb20] Olson, P., Deguen, R., Hinnov, L. A. & Zhong, S. J. (2013). *Phys. Earth Planet. Intl*, **214**, 87–103.

[bb21] Petitgirard, S., Borchert, M., Andrault, D., Appel, K., Mezouar, M. & Liermann, H. P. (2012). *Rev. Sci. Instrum.* **83**, 013904. 10.1063/1.3680573 22299967

[bb22] Pozzo, M., Davies, C., Gubbins, D. & Alfe, D. (2012). *Nature (London)*, **485**, 355–358. 10.1038/nature11031 22495307

[bb23] Salamat, A., Hector, A. L., Gray, B. M., Kimber, S. A. J., Bouvier, P. & McMillan, P. F. (2013). *J. Am. Chem. Soc.* **135**, 9503–9511. 10.1021/ja403368b PMC3715886 23721167

[bb24] Salamat, A., Woodhead, K., McMillan, P. F., Cabrera, R. Q., Rahman, A., Adriaens, D., Cora, F. & Perrillat, J. P. (2009). *Phys. Rev. B*, **80**, 104106.

[bb25] Santoro, M., Gorelli, F. A., Bini, R., Haines, J., Cambon, O., Levelut, C., Montoya, J. A. & Scandolo, S. (2012). *Proc. Natl Acad. Sci. USA*, **109**, 5176–5179. 10.1073/pnas.1118791109 PMC3325671 22431594

[bb26] Schultz, E., Mezouar, M., Crichton, W., Bauchau, S., Blattmann, G., Andrault, D., Fiquet, G., Boehler, R., Rambert, N., Sitaud, B. & Loubeyre, P. (2005). *High Press. Res.* **25**, 71–83.

[bb27] Shen, G. Y., Rivers, M. L., Wang, Y. B. & Sutton, S. R. (2001). *Rev. Sci. Instrum.* **72**, 1273–1282.

[bb28] Toby, B. H. (2001). *J. Appl. Cryst.* **34**, 210–213.

[bb29] Young, A. F., Sanloup, C., Gregoryanz, E., Scandolo, S., Hemley, R. J. & Mao, H. K. (2006). *Phys. Rev. Lett.* **96**, 155501. 10.1103/PhysRevLett.96.155501 16712167

[bb30] Zerr, A. & Boehler, R. (1994). *Nature (London)*, **371**, 506–508.

